# Bone transport with unilateral external fixator for humeral defect reconstruction: a retrospective analysis of post-traumatic osteomyelitis

**DOI:** 10.3389/fsurg.2025.1568285

**Published:** 2025-05-07

**Authors:** Yimurang Hamiti, Patiman Abudureyimu, Gang Lyu, Aihemaitijiang Yusufu, Maimaiaili Yushan

**Affiliations:** ^1^ Department of Microrepair and Reconstructive Surgery, The First Affiliated Hospital of Xinjiang Medical University, Urumqi, Xinjiang, China; ^2^ Imaging Center of the First Affiliated Hospital of Xinjiang Medical University, Urumqi, Xinjiang, China; ^3^ Department of Orthopedic Surgery, The Fourth Affiliated Hospital of Xinjiang Medical University, Traditional Chinese Medicine Hospital of Xinjiang Uyghur Autonomous Region, Urumqi, Xinjiang, China

**Keywords:** bone transport, distraction osteogenesis, Ilizarov technique, humeral defect, osteomyelitis

## Abstract

**Background:**

Large humeral defects resulting from post-traumatic osteomyelitis present significant challenges in orthopedic reconstruction. This study evaluates the efficacy of bone transport using a unilateral external fixator for treating such defects.

**Methods:**

A retrospective analysis was conducted on 15 patients (9 males, 6 females; mean age 29.3 ± 11.7 years) with humeral defects due to post-traumatic osteomyelitis, treated between January 2017 and January 2021. The mean defect size was 7.2 ± 1.4 cm (range, 5.3–9.8 cm). All patients underwent bone transport using a unilateral external fixator. Primary outcomes included external fixation time (EFT), external fixation index (EFI), and bone healing time. Secondary outcomes comprised ASAMI bone and functional scores, DASH scores, range of motion, and complications.

**Results:**

The mean EFT was 342.5 ± 35.6 days (range, 290–410 days), and the mean EFI was 47.5 ± 3.8 days/cm (range, 42–54 days/cm). Bone union was achieved in all cases, with a mean healing time of 11.4 ± 1.2 months (range, 9.5–13.5 months). ASAMI bone results were excellent in 10 patients (66.7%) and good in 4 (26.7%). ASAMI functional results showed excellent outcomes in 8 patients (53.3%) and good in 5 (33.3%). The mean DASH score improved significantly from 35.2 ± 3.6 preoperatively to 15.5 ± 3.2 at final follow-up (*P* < 0.001). Complications occurred in 7 patients (46.7%), with pin site infections being the most common (26.7%).

**Conclusion:**

Bone transport using a unilateral external fixator demonstrates efficacy in treating large humeral defects due to post-traumatic osteomyelitis, yielding good to excellent bone and functional outcomes with manageable complications. These findings extend previous research, highlighting the technique's efficacy in achieving both bone union and functional restoration. This approach deserves consideration as a primary treatment option for complex humeral reconstruction cases, particularly when traditional methods may be insufficient.

## Introduction

Humeral bone defects pose significant challenges in orthopedic surgery, often resulting from trauma, infection, tumor resection, or failed internal fixation (1, 2). These defects can lead to substantial functional impairment and disability if not adequately addressed. Traditional treatment options for large humeral defects include acute shortening, compression-distraction at the fracture site, shortening followed by lengthening at a distant corticotomy site, and vascularized or non-vascularized fibular grafting (3). However, these methods have limitations, including functional compromise with isolated shortening, timing challenges with compression-distraction, and technical complexity and donor site morbidity associated with fibular grafting.

Bone transport, a technique based on the principles of distraction osteogenesis, has emerged as a promising alternative for managing large bone defects (4). This method, pioneered by Ilizarov, allows for gradual regeneration of bone tissue through the controlled distraction of a surgically created osteotomy (5). While bone transport has been widely applied in the lower extremities, its use in the humerus has been less extensively reported (6).

The humerus presents unique considerations for bone transport compared to lower limb applications. Unlike weight-bearing bones, the humerus does not benefit from axial loading during the consolidation phase, which may affect the quality of regenerate bone (7). Additionally, the proximity of neurovascular structures, particularly the radial nerve, necessitates careful surgical planning and execution (8). However, the humerus has demonstrated favorable characteristics for distraction osteogenesis, including a higher rate of callus formation compared to the tibia and potentially faster functional recovery (9).

Various external fixation devices have been employed for humeral bone transport, including circular frames and monolateral fixators (10, 11). While circular frames offer multi-planar stability, they can be cumbersome and poorly tolerated in the upper extremity. Monolateral fixators have gained popularity due to their lower profile and patient comfort, but their ability to provide adequate stability for humeral reconstruction has been debated (12).

Recent studies have reported encouraging outcomes with humeral bone transport. Kiran et al. demonstrated successful treatment of humeral nonunions using the Ilizarov technique, with good functional results and manageable complications (13). Despite these promising results, humeral bone transport remains understudied.

The present case series aims to contribute to the growing body of knowledge on humeral bone transport by reporting our experience with a series of patients treated using a monolateral external fixator. We hypothesize that this technique can effectively address large humeral defects while maintaining a favorable complication profile. By analyzing our results and comparing them to existing literature, we hope to provide insights into the nuances of humeral bone transport and identify areas for future research and technique refinement. In summary, while bone transport has shown promise in managing humeral defects, many questions remain regarding its optimal application in the upper extremity. This study seeks to address these knowledge gaps and further elucidate the role of bone transport in the armamentarium of techniques for humeral reconstruction.

## Patients and methods

### Study design and patient selection

This retrospective case series study was conducted at a single center between January 2017 and January 2021. The primary objective was to examine patients who underwent bone transport for large humeral defects resulting from post-traumatic osteomyelitis. To ensure consistency in technique, all procedures were performed by the same senior orthopedic surgeon. The study protocol was approved by the Institutional Ethics Committee of our institute, which waived the requirement for informed consent due to the retrospective nature of the study.

Inclusion criteria encompassed: (1) Patients with humeral defects greater than 5 cm following radical debridement for post-traumatic osteomyelitis; (2) Treatment via bone transport using a unilateral external fixator; (3) Minimum follow-up duration of 2 years post-surgery; (4) Patients of all ages, including pediatric cases. Exclusion criteria included: (1) Humeral defects from other etiologies (e.g., primary bone tumors, congenital defects); (2) Severe osteoporosis; (3) Uncontrolled diabetes mellitus (HbA1c >8%); (4) Severe peripheral vascular disease affecting the upper limb; (5) Active malignancy; (6) Insufficient follow-up data.

The diagnosis of post-traumatic osteomyelitis was based on a combination of clinical presentation (persistent pain, swelling, discharge), laboratory findings (elevated erythrocyte sedimentation rate, C-reactive protein, and white blood cell count), and imaging studies (plain radiographs, magnetic resonance imaging, and/or computed tomography). All eligible patients were identified through a systematic review of our institution's electronic medical records. The medical records were then manually reviewed to confirm eligibility based on the inclusion and exclusion criteria. The bone defect size was measured on standardized anteroposterior and lateral radiographs, taken with the arm in neutral rotation. The measurement was performed independently by two experienced orthopedic surgeons, and the average value was used for analysis. In cases where the measurements differed by more than 5 mm, a third observer was consulted to reach a consensus. All patients were treated using a standardized surgical technique and postoperative protocol. The follow-up period was calculated from the date of external fixator removal to the last clinical and radiographic evaluation.

### Preoperative evaluation and planning

All patients underwent a comprehensive preoperative evaluation to assess their overall health status, the extent of the humeral defect, and the severity of osteomyelitis. This evaluation began with a thorough history, focusing on the initial injury, previous treatments, and the course of infection. A detailed physical examination was conducted, which included assessment of arm length discrepancy, range of motion of the shoulder and elbow joints, neurovascular status of the affected limb, and the condition of soft tissues, particularly noting any sinus tracts or areas of inflammation.

Preoperative laboratory tests were performed to assess the severity of infection and the patient's nutritional status. These tests included complete blood count, erythrocyte sedimentation rate (ESR), C-reactive protein (CRP), serum albumin, and culture and sensitivity tests of any discharging sinuses.

Imaging studies played a crucial role in preoperative planning. Plain radiographs (anteroposterior and lateral views) of the entire humerus were obtained to evaluate bone quality, defect size, and presence of sequestra. Computed tomography (CT) scans with 3D reconstruction were performed to better delineate the bone defect, assess cortical integrity, and identify any intramedullary sequestra. In select cases, magnetic resonance imaging (MRI) was used to evaluate soft tissue involvement and identify any occult abscesses.

Based on the clinical and radiological evaluation, a detailed preoperative plan was formulated for each patient. This plan included estimation of the extent of debridement required, planning of the corticotomy site (typically in the metaphyseal region for better bone formation), selection of appropriate external fixator configuration, calculation of the expected distraction period and total treatment time, and anticipation of potential complications and their management strategies.

### Surgical technique

All surgeries were performed under general anesthesia with the patient in a supine position. The affected upper limb was prepared and draped in a sterile fashion, ensuring full access to the entire humerus. The first step involved thorough debridement of the infected area. A longitudinal incision was made over the previous surgical site, taking care to preserve any viable soft tissue. All necrotic bone, infected soft tissue, and any implants from previous surgeries were meticulously removed. The medullary canal was opened both proximally and distally, and reamed to remove any residual infected tissue. Multiple tissue samples were obtained for microbiological analysis. The wound was then copiously irrigated with saline solution containing gentamicin.

Following debridement, the unilateral external fixator (Orthofix LRS, Shanghai CIIC Medical Instrument Co., Ltd.) was applied. The first pin was inserted proximally, perpendicular to the long axis of the humerus and centrally in both anteroposterior and lateral planes. The second pin was placed distally, above the olecranon fossa, again ensuring central placement. The external fixator was then mounted on these two pins, and additional pins were inserted using the fixator as a guide. Care was taken to avoid the radial nerve during pin insertion, particularly in the middle third of the humerus.

The corticotomy was performed using a minimally invasive subperiosteal technique. A small incision, approximately 1 cm in length, was made over the planned osteotomy site. The periosteum was carefully elevated using a periosteal elevator, taking care to preserve its integrity. Under fluoroscopic guidance, multiple drill holes were made in a transverse plane through a single cortex. These holes were then connected using a thin osteotome, creating a partial cortical breakage. The osteotomy was completed by manually breaking the opposite corte. The completion of the corticotomy was confirmed under fluoroscopy, ensuring that the bone was completely divided while the surrounding soft tissues, particularly the periosteum, remained intact. This minimally invasive approach helps to maintain the biological environment necessary for successful bone transport while minimizing soft tissue trauma.

### Postoperative management

Distraction phase commenced on the seventh postoperative day, following the latency period recommended by Ilizarov. The distraction rate was initially set at 0.25 mm four times daily, totaling 1 mm per day. This rate was adjusted based on the quality of regenerate bone formation as assessed on weekly radiographs. Patients were instructed on proper pin site care, which included daily cleaning with normal saline and application of chlorhexidine-soaked gauze. They were also educated about the signs of pin site infection and the importance of maintaining good hygiene. Active and passive range of motion exercises for the shoulder and elbow joints were initiated from the first postoperative day to prevent joint stiffness. Patients were encouraged to use the affected limb for light daily activities as tolerated. Prophylactic antibiotics were administered for 24 h postoperatively. For patients with active infection, culture-specific antibiotics were continued for 6 weeks, with regular monitoring of inflammatory markers (ESR and CRP).

### Follow-up and outcome measures

Patients were followed up biweekly during the distraction phase and monthly during the consolidation phase at our outpatient clinic. Each follow-up visit included a comprehensive clinical examination, radiographic evaluation, and when necessary, laboratory tests. Clinical examination focused on pin site condition, joint range of motion, neurovascular status, and pain assessment. Radiographic evaluation consisted of anteroposterior and lateral views to assess bone transport progress, regenerate quality, and overall limb alignment. Adjustments to the rate and rhythm of distraction were made based on the radiographic evaluation of each distraction site and patient tolerance (1). The external fixator was removed following a dynamization period of one month, which commenced once the transferred bone segment reached the docking site and a minimum of three bridging calluses were visible on both anteroposterior and lateral radiographs (14). The duration from frame application to removal, termed external fixation time (EFT), was recorded in days. The external fixation index (EFI) was calculated by dividing the EFT by the length of the regenerated bone (days/cm) (15).

Standardized radiographic evaluation was performed using digital anteroposterior and lateral radiographs obtained at consistent intervals. All radiographs were calibrated using the known diameter of the fixator rods as reference to ensure accurate measurements. Regenerate quality was assessed based on the classification proposed by Li et al., categorizing regenerate as “good” (homogeneous cloud-like appearance with at least three visible cortices), “fair” (heterogeneous appearance with at least two visible cortices), or “poor” (sparse or irregular mineralization with fewer than two visible cortices). The absence of pain at the docking site during clinical stress testing was used as a supplementary criterion for union. Two experienced orthopedic surgeons independently evaluated all radiographs.

The outcome measures included: (1) Bone healing time: defined as the time from the start of distraction to radiographic evidence of consolidation (presence of at least three cortices on orthogonal radiographs) (14); (2) Association for the Study and Application of Methods of Ilizarov (ASAMI) scoring system: Used to evaluate bone and functional results (16). The bone results were classified according to the following predefined criteria: Excellent: Union achieved with no infection, deformity <7°, and limb length discrepancy <2.5 cm; Good: Union achieved with any two of the following: absence of infection, deformity <7°, limb length discrepancy <2.5 cm; Fair: Union achieved with any one of the following: absence of infection, deformity <7°, limb length discrepancy <2.5 cm; Poor: Nonunion or refracture, or none of the following: absence of infection, deformity <7°, limb length discrepancy <2.5 cm. Functional results were assessed based on five predetermined criteria, with results classified as: Excellent: Active, no limp, minimum joint stiffness (loss of <15° shoulder or elbow motion), no reflex sympathetic dystrophy (RSD), and insignificant pain; Good: Active, with one or two of the following: limp, joint stiffness (loss of 15°–30° shoulder or elbow motion), RSD, or significant pain; Fair: Active, with three or all of the following: limp, joint stiffness (loss of >30° shoulder or elbow motion), RSD, or significant pain; Poor: Inactive (unemployment or inability to perform daily activities due to injury). (3) Functional outcomes: assessed using the Disabilities of the Arm, Shoulder and Hand (DASH) score at the final follow-up (17). This 30-item self-reported questionnaire measures physical function and symptoms in people with musculoskeletal disorders of the upper limb, with scores ranging from 0 (no disability) to 100 (most severe disability). To minimize assessment bias, all functional evaluations were performed by two independent observers not directly involved in the surgical procedures, with any disagreements resolved by consensus discussion. (4) Range of motion of the shoulder and elbow joints. (5) Time to return to work or daily activities, recorded in weeks from the date of frame removal. (6) Patient satisfaction, evaluated at final follow-up using a Visual Analog Scale (VAS). (7) Complications encountered during treatment were meticulously recorded and categorized as per the Paley classification, which differentiates between problems, obstacles, and true complications (18). This classification system distinguishes a “problem” as an issue resolved by the end of treatment without surgical intervention, an “obstacle” as a complication resolved surgically by treatment conclusion, and a “true complication” as a persistent issue during the posttreatment period.

### Statistical analysis

All statistical analyses were performed using SPSS version 26.0 (IBM Corp., Armonk, NY, USA). The Shapiro–Wilk test was used to assess the normality of continuous variables. Descriptive statistics were presented as mean ± standard deviation (SD) with ranges for continuous variables and as frequencies with percentages for categorical variables. For comparing preoperative and postoperative continuous variables (DASH scores, shoulder and elbow range of motion), paired *t*-tests were used for normally distributed data. The level of statistical significance was set at *P* < 0.05.

## Results

**Figure 1:**
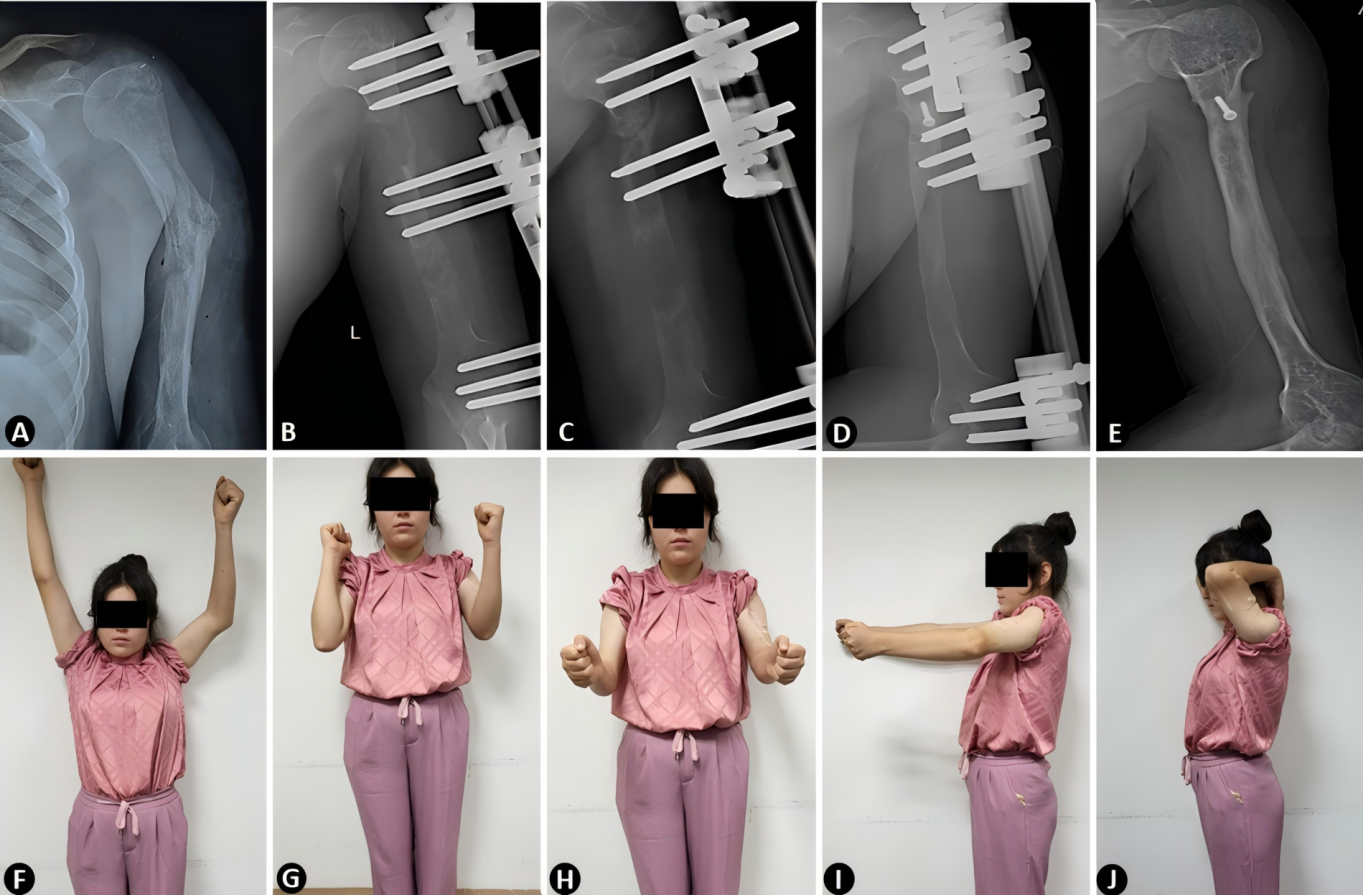
Preoperative and postoperative radiographic and clinical outcomes of humeral defect reconstruction using unilateral external fixator-assisted bone transport. **(A)** Preoperative radiograph illustrating post-traumatic osteomyelitis. **(B–D)** Sequential postoperative radiographs demonstrating the progression of bone transport during the distraction and consolidation phases, with gradual bone regeneration and alignment. **(E)** Final radiograph confirming complete bone union and anatomical restoration of the humerus. **(F–J)** Clinical photographs at the final follow-up showcasing functional recovery. The patient achieved satisfactory functional outcomes with restored limb use in daily activities and minimal complications.

A total of 15 patients (9 males, 6 females) with a mean age of 29.3 ± 11.7 years (range, 12–55 years) were included in this study. The mean defect length was 7.2 ± 1.4 cm (range, 5.3–9.8 cm). The demographic data and bone defect characteristics are summarized in Table 1. Typical case is shown in Figure 1.

**Table 1:** Patient demographics and bone defect characteristics.

Characteristic	Value
Number of patients	15
Gender (male/female)	9/6
Age (years), mean ± SD (range)	29.3 ± 11.7 (12–55)
Bone defect size (cm), mean ± SD (range)	7.2 ± 1.4 (5.3–9.8)

The treatment parameters and clinical outcomes are presented in Table 2. The mean external fixation time (EFT) was 342.5 ± 35.6 days (range, 290–410 days), and the mean external fixation index (EFI) was 47.5 ± 3.8 days/cm (range, 42–54 days/cm). The average bone healing time was 11.4 ± 1.2 months (range, 9.5–13.5 months). The mean time to return to work or daily activities was 24.3 ± 3.6 weeks (range, 20–32 weeks). Patient satisfaction, as measured by VAS, averaged 7.8 ± 0.9 (range, 6–9).

**Table 2:** Treatment outcomes and bone transport parameters.

Parameter	Value
External fixation time (days), mean ± SD (range)	342.5 ± 35.6 (290–410)
External fixation index (days/cm), mean ± SD (range)	47.5 ± 3.8 (42–54)
Bone healing time (months), mean ± SD (range)	11.4 ± 1.2 (9.5–13.5)
Time to return to work/daily activities (weeks), mean ± SD (range)	24.3 ± 3.6 (20–32)
Patient satisfaction (VAS 0–10), mean ± SD (range)	7.8 ± 0.9 (6–9)

Analysis of radiographic progression across our cohort revealed distinct patterns in regenerate formation and consolidation. Initial regenerate was typically visible on radiographs between 3 and 6 weeks post-corticotomy. Among the 15 cases, we observed that regenerate quality could be classified as “good” in 9 cases, “fair” in 5 cases, and “poor” in 1 case. The speed of regenerate maturation demonstrated a notable correlation with patient age, with younger patients showing more rapid and robust callus formation compared to older patients. The quality of regenerate also appeared to influence the overall treatment duration, with cases demonstrating good-quality regenerate generally achieving shorter external fixation times proportional to their defect sizes.

The comparison of functional outcomes between preoperative and final follow-up is shown in Table 3. The DASH score improved significantly from 35.2 ± 3.6 (range, 28–42) preoperatively to 15.5 ± 3.2 (range, 8–22) at final follow-up (*P* < 0.001). Functional recovery followed a relatively predictable pattern, with early improvements in elbow motion (typically regaining 70% of final range within 3 months of frame removal) followed by more gradual improvements in shoulder function. By final follow-up, shoulder flexion increased from 82.5° ± 10.8° (range, 65°–95°) to 135°.5 ± 12.5° (range, 110–150°) (*P* < 0.001), while elbow flexion improved from 80.5 ± 12.2° (range, 60°–95°) to 80.5° ± 12.2° (range, 60°–95°) (*P* < 0.001). Larger defect sizes were associated with greater residual functional limitations, particularly in shoulder abduction and external rotation.

**Table 3:** Comparison of functional outcomes.

Parameter	Preoperative	Final follow-up	*P*-value
DASH score, mean ± SD (range)	35.2 ± 3.6 (28–42)	15.5 ± 3.2 (8–22)	<0.001
Shoulder flexion (degrees), mean ± SD (range)	82.5 ± 10.8 (65–95)	135.5 ± 12.5 (110–150)	<0.001
Elbow flexion (degrees), mean ± SD (range)	80.5 ± 12.2 (60–95)	125.2 ± 11.5 (105–140)	<0.001

According to the ASAMI classification (Table 4), the bone results showed 10 patients (66.7%) rated as excellent, 4 (26.7%) as good, and 1 (6.6%) as fair. The functional results revealed 8 patients (53.3%) as excellent, 5 (33.3%) as good, and 2 (13.3%) as fair. No poor results were observed in either category.

**Table 4:** ASAMI bone and functional results.

Result category	Bone results, *n* (%)	Functional results, *n* (%)
Excellent	10 (66.7%)	8 (53.3%)
Good	4 (26.7%)	5 (33.3%)
Fair	1 (6.6%)	2 (13.3%)
Poor	0 (0%)	0 (0%)

Complications occurred in 7 patients (46.7%). According to Paley's classification (Table 5), there were 4 problems (pin site infections resolved with oral antibiotics), 2 obstacles (1 premature consolidation requiring repeat corticotomy, 1 delayed union at the docking site requiring bone grafting), and 1 true complication (transient radial nerve palsy). The radial nerve palsy occurred during the treatment and resolved completely without surgical intervention.

**Table 5:** Complications according Paley criteria.

Complication	Number of patients (%)
Problems	
Pin site infections	4 (26.7%)
Obstacles	
Premature consolidation	1 (6.7%)
Delayed union at docking site	1 (6.7%)
True complications	
Transient radial nerve palsy	1 (6.7%)
Total complications	7 (46.7%)

## Discussion

This case series demonstrates that bone transport using a unilateral external fixator is an effective method for treating large humeral defects resulting from post-traumatic osteomyelitis. Our study of 15 patients showed satisfactory bone and functional outcomes, with manageable complications. These results contribute to the growing body of evidence supporting the use of distraction osteogenesis in upper limb reconstruction, an area that has received less attention compared to lower limb applications.

The mean external fixation time (EFT) of 342.5 ± 35.6 days and external fixation index (EFI) of 47.5 ± 3.8 days/cm in our study are comparable to those reported in previous literature, albeit slightly longer. Our slightly higher EFI might be attributed to the larger defect sizes in our series (mean 7.2 ± 1.4 cm). However, our results are still within the acceptable range for upper limb reconstruction. The longer EFI observed in humeral reconstruction compared to lower limb procedures raises interesting questions about the biology of distraction osteogenesis in different anatomical locations. Ilizarov's original work primarily focused on the lower limb, where axial loading plays a crucial role in stimulating bone formation (5). The upper limb, being non-weight bearing, lacks this mechanical stimulus, which may contribute to the slower rate of bone formation and consolidation (19).

This relatively prolonged treatment duration represents a significant burden for patients and highlights the need for strategies to optimize the bone transport process in humeral applications. Several approaches could potentially enhance bone formation and reduce treatment time in future applications. Biological augmentation represents a promising avenue for accelerating bone regeneration. Growth factors such as bone morphogenetic proteins (BMPs), platelet-rich plasma (PRP), or autologous bone marrow aspirate concentration (BMAC) applied at the corticotomy site might enhance initial regenerate formation. Dehghan et al. have demonstrated that local application of BMP-7 accelerated bone regeneration in experimental models of distraction osteogenesis (20). However, the cost-effectiveness and safety profile of these interventions require careful consideration. Mechanical stimulation methods may also promote bone formation. While the upper limb lacks the natural axial loading that benefits lower limb distraction osteogenesis, alternative forms of mechanical stimulation such as low-intensity pulsed ultrasound (LIPUS) or pulsed electromagnetic field therapy (PEMF) could potentially enhance regenerate maturation. These non-invasive modalities have shown promise in fracture healing and may be particularly valuable in non-weight-bearing applications. Optimization of distraction protocols based on individual patient characteristics represents another potential strategy. Younger patients typically demonstrate more robust regenerate formation and might tolerate more rapid distraction rates, while older patients or those with comorbidities affecting bone metabolism might benefit from more conservative protocols with longer consolidation periods. Radiographic monitoring with protocol adjustments based on regenerate quality could allow for patient-specific optimization. Hybrid approaches combining external fixation during the distraction phase with conversion to internal fixation after docking could potentially reduce external fixation time while maintaining stability during consolidation, potentially improving patient comfort and reducing pin site complications during the lengthy consolidation phase. Finally, pharmacological approaches targeting bone metabolism have shown encouraging results in complex fracture healing and might accelerate consolidation in distraction osteogenesis. The cost and potential side effects of these interventions must be balanced against their benefits, particularly in younger patients without underlying bone metabolism disorders. Implementation and rigorous evaluation of these strategies in future studies could potentially reduce the treatment burden associated with humeral bone transport while maintaining the good structural and functional outcomes observed in our series.

Our ASAMI bone results, with 66.7% excellent and 26.7% good outcomes, are comparable to those reported for infected nonunion of the humerus treated with Ilizarov fixation. This suggests that unilateral fixators can achieve similar bone results to circular frames in humeral reconstruction, while potentially offering better patient comfort and easier application (12).

The quality of regenerate bone in the upper limb has been a subject of debate in the literature. Tanaka et al. (7) reported that callus formation in the humerus during limb lengthening was more robust compared to the femur and tibia. This observation challenges the conventional wisdom that weight-bearing is essential for optimal bone formation. It raises the possibility that other factors, such as the rich vascular supply of the upper limb or differences in the local stem cell population, may compensate for the lack of axial loading.

However, the assessment of bone quality in distraction osteogenesis remains a challenge. While conventional radiographs provide a general impression of bone formation, they lack the ability to quantify bone density and microarchitecture. Advanced imaging techniques such as quantitative computed tomography (qCT) and high-resolution peripheral quantitative computed tomography (HR-pQCT) offer promising avenues for more detailed assessment of regenerate bone quality (21). Future studies incorporating these modalities could provide valuable insights into the structural and biomechanical properties of regenerate bone in the humerus.

The functional outcomes in our series, as measured by the ASAMI functional results (53.3% excellent, 33.3% good), are promising. The DASH score improving significantly from 35.2 ± 3.6 to 15.5 ± 3.2 (*P* < 0.001). The improvement in shoulder flexion from 82.5° to 135.5° and elbow flexion from 80.5° to 80.5° demonstrates that joint function can be well preserved during the bone transport process. These results underscore the potential of bone transport to not only address structural deficits but also restore meaningful function in complex upper limb injuries. However, these measurements have limitations in fully capturing the multidimensional aspects of recovery, including psychological adaptation, return to occupation, and patient satisfaction. The residual DASH score of 15.5 suggests that while patients achieved substantial improvement, some functional limitations likely persisted at final follow-up. Although our study did not employ specific quality of life instruments or detailed occupational assessments, the functional parameters measured suggest that most patients would have been capable of returning to non-manual or light manual work following reconstruction. The range of motion improvements, particularly in shoulder flexion (from 82.5° to 135.5°) and elbow flexion (from 80.5° to 125.2°), indicate recovery of essential upper limb functions necessary for most daily activities. Future studies would benefit from incorporating standardized quality of life measures, detailed occupational assessments, and patient satisfaction ratings to provide more comprehensive evaluation of treatment outcomes beyond traditional clinical parameters. Additionally, longer follow-up periods would provide valuable insights into the durability of functional improvements and the potential for late complications such as refracture or progressive joint stiffness.

The optimal rehabilitation strategy for patients undergoing humeral bone transport remains to be defined. While our results support early mobilization, questions remain regarding the ideal timing, intensity, and progression of exercises. The balance between promoting joint mobility and protecting the regenerate bone is delicate and may require individualized approaches based on patient factors and the characteristics of the bone defect (22). Moreover, the role of concurrent soft tissue management in optimizing functional outcomes deserves attention. In cases of post-traumatic osteomyelitis, the surrounding soft tissues are often compromised, which can impact joint mobility and overall function. Techniques such as soft tissue lengthening and targeted tendon transfers may play a crucial role in maximizing functional recovery (23).

The complication rate in our series (46.7%) is within the range reported in the literature for complex limb reconstruction procedures. While this rate may seem high, it is important to contextualize it within the challenging nature of these cases and the prolonged treatment course.

Pin site infections, the most common complication in our series (26.7%), remain a persistent challenge in external fixation. Recent research has focused on strategies to reduce pin site infections, including the use of hydroxyapatite-coated pins, silver-coated pins, and local antibiotic delivery systems. A systematic review by Kazmers et al. (24) suggested that chlorhexidine-based pin site care may be superior to other regimens in preventing infections. Our protocol, which included daily cleaning with normal saline and application of chlorhexidine-soaked gauze, aligns with these recommendations. The low incidence of major complications in our series, particularly the single case of transient radial nerve palsy (6.7%), is encouraging. However, it highlights the need for meticulous surgical technique and careful monitoring during the distraction phase. The proximity of the radial nerve to the surgical field in humeral procedures necessitates a thorough understanding of the regional anatomy and potential variations.

The inclusion of pediatric patients in our series, with the youngest being 12 years old, raises important considerations regarding the application of distraction osteogenesis in skeletally immature individuals. While our results suggest that bone transport can be safely applied in this population, several unique factors must be considered.

The growth potential of the physis in pediatric patients presents both opportunities and challenges. On one hand, the robust healing capacity of young bone may facilitate faster consolidation and remodeling. On the other hand, there is a risk of growth disturbance if the physis is inadvertently injured during the procedure. Careful planning of the osteotomy site and pin placement is crucial to avoid physeal injury. Furthermore, the psychological impact of prolonged external fixation on pediatric patients should not be underestimated. Strategies to minimize the psychosocial burden of treatment, such as involving child life specialists and providing peer support, may be particularly important in this population.

Our use of a unilateral fixator for humeral bone transport contributes to the ongoing debate regarding the optimal fixation method for distraction osteogenesis. Our choice of unilateral external fixators for humeral bone transport deserves specific discussion. While circular frames have traditionally been preferred for complex limb reconstruction due to their superior multiplanar stability (25), several considerations led to our selection of unilateral fixators for this specific application. From a biomechanical perspective, the non-weight-bearing function of the upper limb reduces the importance of axial and torsional loading compared to lower limb applications. Unilateral fixators, when properly applied with adequate pin spread and optimal positioning, provide sufficient stability for humeral reconstruction while offering several distinct advantages. Patient comfort and compliance considerations strongly influenced our decision, as the smaller profile and reduced bulk of unilateral fixators are particularly beneficial in the upper extremity where bulky circular frames can significantly impede activities of daily living, personal hygiene, and clothing options. The psychological burden of treatment, already substantial given the lengthy external fixation times required, may be reduced with the less conspicuous unilateral devices. From a technical standpoint, unilateral fixators allow for simpler application, adjustment, and removal, which can reduce operative time and potential complications. The reduced number of pins and soft tissue penetration may decrease the risk of neurovascular injury - a particular concern in the humerus given the proximity of the radial nerve. Additionally, the unilateral configuration facilitates easier soft tissue management and pin site care, potentially contributing to the relatively low rate of major complications observed in our series. Patient-specific factors also influenced fixator selection. Cases with significant angular or rotational deformities in addition to bone loss might benefit more from the multiplanar correctional capabilities of circular frames. However, our cohort primarily presented with straight-segment defects following debridement, making the unilateral configuration suitable. Similarly, cases with extensive soft tissue loss requiring flap coverage might benefit from the circumferential access provided by circular frames, but our patients had adequate soft tissue envelopes following debridement and infection control. Future comparative studies, ideally randomized controlled trials, could provide valuable insights into the relative merits of these fixation methods in humeral bone transport.

Our study has several limitations that should be acknowledged. First, as a retrospective case series, it lacks a control group for direct comparison. This design limitation significantly impacts our ability to definitively establish whether unilateral external fixation offers superior, equivalent, or inferior outcomes compared to alternative reconstruction methods such as circular frames, vascularized fibular grafts, or induced membrane techniques. The absence of a comparative group introduces potential selection bias, as patients with more complex deformities or severe soft tissue compromise might have been treated with other methods. Without direct comparison, the relative benefits and drawbacks of our approach in terms of treatment duration, complication rates, and functional outcomes remain uncertain. Second, our sample size, while reasonable for this specialized procedure, limits our ability to perform robust subgroup analyses or identify rare complications. The relatively small number of patients also reduces the statistical power to identify potential predictors of better or worse outcomes. Third, the involvement of a single experienced surgeon at a single center raises questions about the generalizability of our results. The learning curve associated with bone transport and external fixation techniques is steep, and results may vary significantly based on surgeon experience and institutional protocols. Fourth, our follow-up period, while sufficient to assess bone healing and initial functional outcomes, may not capture very long-term complications or functional changes that could emerge over years rather than months. The durability of reconstructed bone and potential for late deformity, refracture, or functional deterioration requires longer-term surveillance. Future research directions should include prospective comparative studies to directly evaluate different reconstruction methods for humeral defects. Ideally, such studies would incorporate randomization when clinically appropriate, include detailed quality of life assessments, and feature longer follow-up periods. Multicenter collaboration would enhance generalizability and increase sample size for more robust statistical analysis of outcomes and predictive factors. Despite these limitations, our results provide valuable insights into the efficacy and safety of unilateral external fixator-assisted bone transport for humeral defect reconstruction, offering a foundation for future comparative studies and technical refinement.

## Conclusion

This study demonstrates that bone transport using a unilateral external fixator is an effective and viable method for treating large humeral defects resulting from post-traumatic osteomyelitis. Our findings reveal good to excellent bone and functional outcomes, with a manageable complication profile. The technique offers a valuable option in the challenging field of upper limb reconstruction, particularly in cases where traditional methods may be insufficient. As our understanding of distraction osteogenesis in the upper limb continues to evolve, we anticipate further refinements in technique, improvements in outcomes, and potential expansion of indications for this versatile approach. The complex nature of humeral reconstruction presents ongoing challenges that serve as a catalyst for innovation, pushing the boundaries of our ability to restore both form and function in complex upper limb injuries. Future prospective studies with larger cohorts and longer follow-up periods are warranted to further validate these findings and explore the long-term outcomes of this promising technique.

## Data Availability

The original contributions presented in the study are included in the article/Supplementary Material, further inquiries can be directed to the corresponding authors.

## References

[1] Akbulut D Coskun M Mirzazada J Sezgic AB. Case report: plate-assisted bone transport with uniplanar external fixator in large bone defects of the humerus. Int J Surg Case Rep. (2024) 120:109898. 10.1016/j.ijscr.2024.109898 38889518 PMC11231522

[2] Jia Q Liu Y Alimujiang A Guo J Chen D Wang Y Nine-year-long complex humeral nonunion salvaged by distraction osteogenesis technique: a case report and review of the literature. BMC Surg. (2022) 22:77. 10.1186/s12893-022-01524-z 35241038 PMC8892714

[3] Jupiter JB von Deck M. Ununited humeral diaphyses. J Shoulder Elbow Surg. (1998) 7:644–53. 10.1016/s1058-2746(98)90016-7 9883429

[4] Borzunov DY. Long bone reconstruction using multilevel lengthening of bone defect fragments. Int Orthop. (2012) 36:1695–700. 10.1007/s00264-012-1562-1 22581353 PMC3535043

[5] Ilizarov GA. The tension-stress effect on the genesis and growth of tissues. Part I. The influence of stability of fixation and soft-tissue preservation. Clin Orthop Relat Res. (1989) 238:249–81. 10.1097/00003086-198901000-00038 2910611

[6] Hosny GA. Humeral lengthening and deformity correction. J Child Orthop. (2016) 10:585–92. 10.1007/s11832-016-0789-6 27826910 PMC5145839

[7] Tanaka K Nakamura K Matsushita T Horinaka S Kusaba I Kurokawa T. Callus formation in the humerus compared with the femur and tibia during limb lengthening. Arch Orthop Trauma Surg. (1998) 117:262–4. 10.1007/s004020050242 9581257

[8] Rozbruch SR Fryman C Bigman D Adler R. Use of ultrasound in detection and treatment of nerve compromise in a case of humeral lengthening. HSS J. (2011) 7:80–4. 10.1007/s11420-010-9182-z 22294962 PMC3026108

[9] Kiss S Pap K Vízkelety T Terebessy T Balla M Szoke G. The humerus is the best place for bone lengthening. Int Orthop. (2008) 32:385–8. 10.1007/s00264-007-0327-8 17323094 PMC2323419

[10] Cattaneo R Catagni MA Guerreschi F. Applications of the Ilizarov method in the humerus. Lengthenings and nonunions. Hand Clin. (1993) 9:729–39. 10.1016/S0749-0712(21)01023-4 8300742

[11] Janovec M. Short humerus: results of 11 prolongations in 10 children and adolescents. Arch Orthop Trauma Surg. (1991) 111:13–5. 10.1007/BF00390185 1772719

[12] Pawar AY McCoy TH Fragomen AT Rozbruch SR. Does humeral lengthening with a monolateral frame improve function? Clin Orthop Relat Res. (2013) 471:277–83. 10.1007/s11999-012-2543-9 22926491 PMC3528891

[13] Kiran M Jee R. Ilizarov’s method for treatment of nonunion of diaphyseal fractures of the humerus. Indian J Orthop. (2010) 44:444–7. 10.4103/0019-5413.69319 20924488 PMC2947734

[14] Fischgrund J Paley D Suter C. Variables affecting time to bone healing during limb lengthening. Clin Orthop Relat Res. (1994) 301:31–7. 8156692

[15] Nakamura K Matsushita T Mamada K Okazaki H Ou W Okuma Y Changes of callus diameter during axial loading and after fixator removal in leg lengthening. Arch Orthop Trauma Surg. (1998) 117:464–7. 10.1007/s004020050294 9801783

[16] Kamarudin KI Then JW Yeoh CW. Bone transport using semicircular Ilizarov ring fixator in the treatment of the infected nonunion of the humerus: a case report. Int E-J Sci Med Educ. (2018) 12(2):12–6. 10.56026/imu.12.2.34

[17] Hudak PL Amadio PC Bombardier C. Development of an upper extremity outcome measure: the DASH (disabilities of the arm, shoulder and hand) [corrected]. the upper extremity collaborative group (UECG). Am J Ind Med. (1996) 29:602–8. 10.1002/(SICI)1097-0274(199606)29:6<602::AID-AJIM4>3.0.CO;2-L 8773720

[18] Paley D. Problems, obstacles, and complications of limb lengthening by the Ilizarov technique. Clin Orthop Relat Res. (1990) 250:81–104. 2403498

[19] Aronson J. Limb-lengthening, skeletal reconstruction, and bone transport with the Ilizarov method. J Bone Joint Surg Am. (1997) 79:1243–58. 10.2106/00004623-199708000-00019 9278087

[20] Dehghan MM Baghaban Eslaminejad M Motallebizadeh N Ashrafi Halan J Tagiyar L Soroori S Transplantation of autologous bone marrow mesenchymal stem cells with platelet-rich plasma accelerate distraction osteogenesis in a canine model. Cell J. (2015) 17:243–52. 10.22074/cellj.2016.3724 26199903 PMC4503838

[21] Burghardt AJ Buie HR Laib A Majumdar S Boyd SK. Reproducibility of direct quantitative measures of cortical bone microarchitecture of the distal radius and tibia by HR-pQCT. Bone. (2010) 47:519–28. 10.1016/j.bone.2010.05.034 20561906 PMC2926164

[22] Watson JT. Distraction osteogenesis. J Am Acad Orthop Surg. (2006) 14:S168–74. 10.5435/00124635-200600001-00037 17003192

[23] Robert Rozbruch S Weitzman AM Tracey Watson J Freudigman P Katz HV Ilizarov S. Simultaneous treatment of tibial bone and soft-tissue defects with the Ilizarov method. J Orthop Trauma. (2006) 20:197–205. 10.1097/00005131-200603000-00006 16648701

[24] Kazmers NH Fragomen AT Rozbruch SR. Prevention of pin site infection in external fixation: a review of the literature. Strategies Trauma Limb Reconstr. (2016) 11:75–85. 10.1007/s11751-016-0256-4 27174086 PMC4960058

[25] Fragomen AT Rozbruch SR. The mechanics of external fixation. HSS J. (2007) 3:13–29. 10.1007/s11420-006-9025-0 18751766 PMC2504087

